# Determinants of attending antenatal care at least four times in rural Ghana: analysis of a cross-sectional survey

**DOI:** 10.1080/16549716.2017.1291879

**Published:** 2017-01-27

**Authors:** Evelyn Sakeah, Sumiyo Okawa, Abraham Rexford Oduro, Akira Shibanuma, Evelyn Ansah, Kimiyo Kikuchi, Margaret Gyapong, Seth Owusu-Agyei, John Williams, Cornelius Debpuur, Francis Yeji, Vida Ami Kukula, Yeetey Enuameh, Gloria Quansah Asare, Enoch Oti Agyekum, Sheila Addai, Doris Sarpong, Kwame Adjei, Charlotte Tawiah, Junko Yasuoka, Keiko Nanishi, Masamine Jimba, Abraham Hodgson, the Ghana EMBRACE Team

**Affiliations:** ^a^ Social Science Department, Navrongo Health Research Centre, Ghana Health Service, Navrongo, Upper East, Ghana; ^b^ Department of Community and Global Health, The University of Tokyo, Tokyo, Japan; ^c^ Navrongo Health Research Centre, Research and Development Division, Ghana Health Service, Navrongo, Upper East, Ghana; ^d^ Research & Development Division, Ghana Health Service, Accra, Ghana; ^e^ Dodowa Health Research Centre, Research and Development Division, Ghana Health Service, Dodowa, Greater Accra, Ghana; ^f^ Kintampo Health Research Centre, Research and Development Division, Ghana Health Service, Kintampo, Brong Ahafo, Ghana; ^g^ Population Department, Navrongo Health Research Centre, Ghana Health Service, Navrongo, Upper East, Ghana; ^h^ Maternal and Child Health Unit, Dodowa Health Research Centre, Ghana Health Service, Dodowa, Greater Accra, Ghana; ^i^ Maternal and Child Health Unit, Kintampo Health Research Centre, Ghana Health Service, Kintampo, Brong Ahafo, Ghana; ^j^ Ghana Health Service, Accra, Ghana; ^k^ Japan International Cooperation Agency Health Section, Accra, Ghana; ^l^ Dodowa Health Research Centre, Ghana Health Service, Dodowa, Greater Accra, Ghana; ^m^ Dodowa Health Research Centre and Regional Institute for Population Studies, University of Ghana, Accra, Ghana; ^n^ Maternal and Child Health Unit, Kintampo Health Research Centre, Kintampo, Brong Ahafo, Ghana; ^o^ Office of International Academic Affairs, Graduate School of Medicine and Faculty of Medicine, The University of Tokyo, Tokyo, Japan; ^p^ Research and Development Division, Ghana Health Service, Accra, Greater Accra, Ghana

**Keywords:** Maternal mortality, determinants, Ghana, women service utilization, antenatal care

## Abstract

**Background:** Improving maternal health is a global challenge. In Ghana, maternal morbidity and mortality rates remain high, particularly in rural areas. Antenatal care (ANC) attendance is known to improve maternal health. However, few studies have updated current knowledge regarding determinants of ANC attendance.

**Objective:** This study examined factors associated with ANC attendance in predominantly rural Ghana.

**Methods:** We conducted a cross-sectional study at three sites (i.e. Navrongo, Kintampo, and Dodowa) in Ghana between August and September 2013. We selected 1500 women who had delivered within the two years preceding the survey (500 from each site) using two-stage random sampling. Data concerning 1497 women’s sociodemographic characteristics and antenatal care attendance were collected and analyzed, and factors associated with attending ANC at least four times were identified using logistic regression analysis.

**Results:** Of the 1497 participants, 86% reported attending ANC at least four times, which was positively associated with possession of national health insurance (AOR 1.64, 95% CI: 1.14–2.38) and having a partner with a high educational level (AOR 1.64, 95% CI: 1.02–2.64) and negatively associated with being single (AOR 0.39, 95% CI: 0.22–0.69) and cohabiting (AOR 0.57, 95% CI: 0.34–0.97). In site-specific analyses, factors associated with ANC attendance included marital status in Navrongo; marital status, possession of national health insurance, partners’ educational level, and wealth in Kintampo; and preferred pregnancy timing in Dodowa. In the youngest, least educated, and poorest women and women whose partners were uneducated, those with health insurance were more likely to report at least four ANC attendances relative to those who did not have insurance.

**Conclusions:** Ghanaian women with low socioeconomic status were less likely to report at least four ANC attendances during pregnancy if they did not possess health insurance. The national health insurance scheme should include a higher number of deprived women in predominantly rural communities.

## Background

Approximately 303,000 maternal deaths occurred worldwide in 2015 [1]. In the same year, sub-Saharan Africa experienced 201,000 maternal deaths despite a 45% reduction since 1990 [1]. Even with this decline, sub-Saharan Africa alone accounted for 66% of maternal deaths worldwide [1]. Despite the significant number of maternal deaths in sub-Saharan Africa, limited progress has been made toward achieving Millennium Development Goal 5 [1].

**Figure 1.**
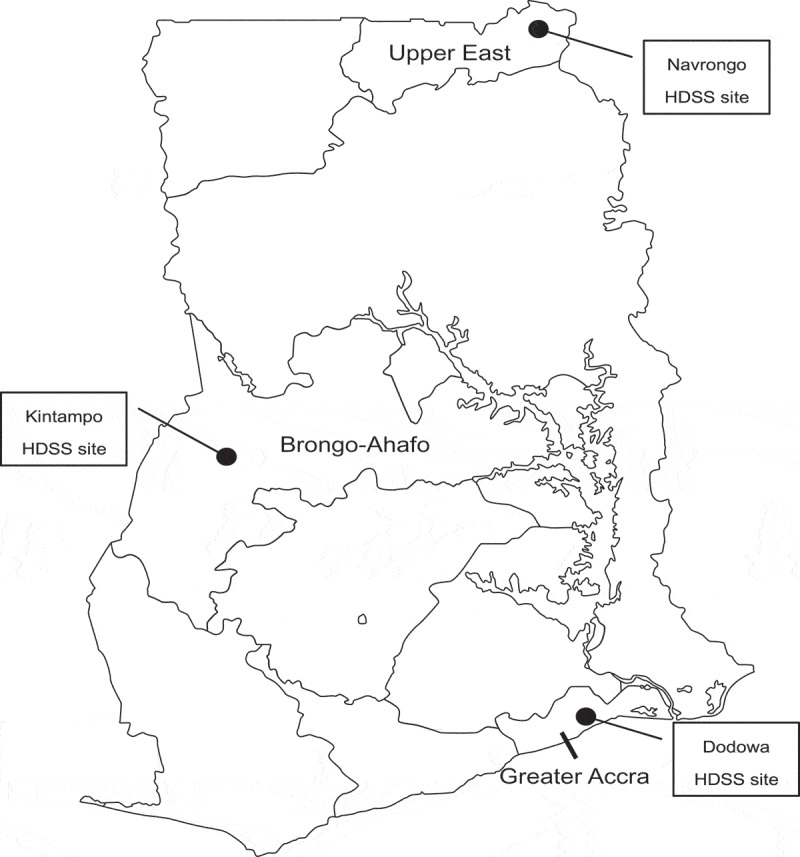
Map of Ghana showing the geographical location of the study sites. Source: http://www2m.biglobe.ne.jp/ZenTech/world/map/Ghana/Outline_Map_of_Ghana.htm.

Professional ANC is crucial in tracking pregnancy to reduce the risks of complications, disability, and death in mothers and their infants [2]. The World Health Organization has advised pregnant women to attend ANC at least four times [3]. Appropriate use of ANC could significantly improve early identification and mitigation of risk factors in pregnancy [4]. In contrast, failure to attend ANC or inadequate ANC attendance could lead to complications, exacerbate pre-existing conditions, or worsen the consequences of an unhealthy lifestyle during pregnancy [5,6].

ANC attendance is influenced by geographical, socioeconomic, religious, cultural, and demographic factors [7–9]. For instance, lower educational levels [9] and socioeconomic factors [2,7] are associated with poor ANC attendance in women in rural areas [2,10]. Spousal support and decisions concerning the use of maternal health services also influence women’s access to skilled care [11]. This could be attributed to the patriarchal system in place in Africa, in which men are usually the heads of households, with the power and authority to make decisions, including those concerning their wives’ use of maternal health services [12–16], for their family members [13]. Religious factors also contribute to women’s decisions regarding ANC attendance [7]. Religion is considered a form of healing and, in some instances, offers interventions for some diseases. In Ghana, some religious groups do not allow modern health intervention and rely mainly on spiritual solutions for health problems [10,17].

In Ghana, maternal, neonatal, and child health are major public health issues. The maternal mortality ratio (MMR) was estimated at 319 per 100,000 live births in 2015, which was higher relative to those of other sub-Saharan African countries [1].

In 2004, the Ghanaian government introduced a policy to ensure free medical care for pregnant women via the Ghana National Health Insurance Scheme (NHIS) [18], which aims to offer women, particularly those in rural areas, the opportunity to seek maternal health services [19]. It includes a comprehensive exemption package that provides ANC, skilled delivery, and postnatal care services [20]. To register as an NHIS subscriber, individuals are interviewed for the purposes of categorization according to two types of registration status: as an annual premium-paying member or a member of the exempt group. Premium-paying members pay a registration fee of GH₵3 (USD 0.63) and a premium of GH₵12 (USD 2.54). Those registering as members of the exempt category (e.g. pregnant women) are required to present proof of pregnancy, such as a current antenatal card; a signed note from a doctor or midwife; a pregnancy test result endorsed by a doctor, midwife, or other prescriber; or an ultrasound scan report, to avoid paying a premium. Membership is renewed annually [21]. The free maternal health services are provided via all public health facilities including the Community-Based Health Planning and Services (CHPS) Program [21], which is a unique primary healthcare system initiated by the Ghanaian government to remove geographical barriers to healthcare [21,22]. The CHPS Program aims to improve survival rates for pregnant women and their children [20,23]. According to the national safe motherhood service protocol, all pregnant women with uncomplicated pregnancies are advised to attend ANC at least four times, with more visits recommended in pregnancies involving complications [24]. In 2014, 87% of women attended ANC at least four times [2]. In Ghana, factors such as age, parity, marital status, wealth, ANC timing, insurance, cost of ANC services, educational level, religion, ethnicity, residence, and transportation have been shown to contribute to women’s decisions regarding ANC attendance [25–28].

However, these studies used data from the Ghana Demographic and Health Survey conducted in 2008. Whether women have benefited from increased access to ANC through the NHIS and CHPS programs since then remains unknown. Therefore, it is vital to update current knowledge regarding ANC attendance and its determinants, as it has important implications for policy developed to ensure ANC attendance by all pregnant women and reduce maternal morbidity and mortality. Therefore, the study examined factors associated with ANC attendance in predominantly rural populations.

## Methods

### Study design and settings

This cross-sectional study was conducted as a situational analysis before starting the intervention of the Ghana Ensure Mothers and Babies Regular Access to Care (EMBRACE) Implementation Research [29] conducted between August and September 2013. This was a collaborative study involving the Ghana Health Service (GHS), the University of Tokyo, and the Japan International Cooperation Agency (JICA). It was implemented at three Health Demographic Surveillance System (HDSS) sites in Navrongo (Upper East Region), Kintampo (Brong Ahafo Region), and Dodowa (Greater Accra Region). The HDSS is used to examine population dynamics in these settings and involves regular collection and processing of information concerning household characteristics, pregnancies, births, deaths, migrations, morbidity, marriages, and vaccination in the districts involved [30].

In Navrongo, the surveillance population numbered approximately 153,000 in 2011 [31]. Navrongo is located in the northern belt of the country, 777 km from the national capital, Accra. With 36 CHPS compounds, the Navrongo area contained a higher number of compounds relative to those recorded for the other two study sites. The program began in Navrongo and spread throughout the Upper East Region. For Kintampo, a surveillance population of approximately 200,000 was reported in 2011 [32]. Kintampo is located in the middle belt of the country, 429 km from Accra, and contains 24 CHPS compounds. For the Dodowa HDSS site, a surveillance population of approximately 115,000 was recorded in 2011. It is approximately 40 km from Accra [33] and contains 20 CHPS compounds.

### Sampling and participant criteria

We used the three HDSS databases to identify women aged 15–49 years who had experienced live birth or stillbirth within the two years preceding the survey. We then used a two-stage random sampling method to select 1500 women (500 from each of the 3 HDSS sites). During the first stage, we randomly selected clusters, or primary sampling units (subdistricts or zones), from the target areas. During the second stage, we randomly selected a specific number of eligible women from each cluster. We included data for 1497 women in the analysis, with data for 3 women excluded because of missing key background information. The inclusion criteria were female sex; reproductive age (15–49 years old); and delivery, including stillbirth, within the two years preceding the survey.

### Data collection

We collected data using a structured questionnaire, which included items concerning social and demographic characteristics such as age, religion, marital status, educational level, partner’s educational level, geographical location (i.e. Navrongo, Kintampo, Dodowa), household assets, national health insurance status, preferred pregnancy timing, ethnicity, religion, and ANC attendance during the pregnancy preceding the most recent birth. We generated a quintile rank for wealth, based on the possession of 18 items representing household assets. In addition, preferred pregnancy timing reflected whether respondents had wished to become pregnant when the pregnancy had occurred, at a later date, or not at all.

The questionnaire was developed in the English language and pretested in communities outside the study sites. The fieldworkers underwent two weeks of training prior to the survey and visited households to interview eligible women.

### Data analysis

We produced descriptive statistics to summarize respondents’ background characteristics. Multivariable logistic regression analysis was performed to identify determinants of attending ANC at least four times across all study sites and at each study site. We determined whether respondents had attended ANC at least four times using the question, ‘How many times did you receive ANC during your last pregnancy?’ The following explanatory variables were analyzed: geographical location, age, marital status, educational level, partner’s educational level, religion, national health insurance status, wealth index, and preferred pregnancy timing. The wealth index consisted of 18 household-related items. These independent variables were selected based on previous studies [25–28]. We generated quintile ranks for wealth status using principal component analysis.

In addition, we performed a chi-square test to examine differences in the proportions of women who reported at least four ANC attendances according to health insurance status in women with low socioeconomic status (i.e. women aged 15–24 years, unmarried women, uneducated women, women whose partners were uneducated, and the poorest women). All *p*- values were two-tailed, and the significance level was set at *p* < 0.05. We performed all statistical analyses using Stata Version 12 (Stata Corp., TX).

### Ethical considerations

We obtained ethical approval for the study from the research ethics committees at the Graduate School of Medicine, The University of Tokyo, and the GHS; the institutional review boards at Navrongo Health Research Centre and Dodowa Health Research Centre; and the institutional ethics committee at Kintampo Health Research Centre. We also obtained written informed consent from all women and the parents/guardians of those aged 15–17 years prior to initiation of the interviews.

## Results

### Respondents’ sociodemographic characteristics

Table 1 shows the characteristics of the 1497 participants from the 3 sites included in the study. The numbers of respondents from Navrongo, Kintampo, and Dodowa were 497, 500, and 500, respectively. In total, 39% of the women and 32% of their partners had received no education. The majority (82%) of the women were aged 15–34 years. More than half of the women were Christians, 24% worshipped ancestral gods, 15% practiced Islam, and 5% identified themselves with other faiths.

**Table 1.** Respondents’ sociodemographic characteristics.

	Navrongo (n = 497)		Kintampo (n = 500)		Dodowa (n = 500)		All sites (N = 1497)	
Characteristics	n	(%)	n	(%)	n	(%)	n	(%)
**Age group**								
15–24	177	(35.6)	162	(33.2)	167	(33.7)	506	(34.2)
25–34	231	(46.5)	218	(44.8)	252	(50.8)	701	(47.4)
35–49	89	(17.9)	107	(22.0)	77	(15.5)	273	(18.4)
**Marital status**								
Married	453	(91.2)	323	(64.6)	134	(26.8)	910	(60.8)
Cohabiting	8	(1.6)	90	(18.0)	297	(59.4)	395	(26.4)
Divorced/widowed/never married	36	(7.2)	87	(17.4)	69	(13.8)	192	(12.8)
**Education**								
None	148	(29.8)	244	(48.8)	192	(38.4)	584	(39.0)
Primary	139	(28.0)	96	(19.2)	109	(21.8)	344	(23.0)
Middle/JSS/JHS	138	(27.8)	138	(27.6)	148	(29.6)	424	(28.3)
Secondary and above	72	(14.4)	22	(4.4)	51	(10.2)	145	(9.7)
**Partner’s education**								
None	165	(33.7)	182	(41.3)	86	(19.6)	433	(31.6)
Primary	92	(18.8)	32	(7.2)	70	(15.9)	194	(14.2)
Middle/JSS/JHS	103	(21.0)	136	(30.8)	180	(41.0)	419	(30.6)
Secondary/SSS/SHS	76	(15.5)	70	(15.9)	75	(17.1)	221	(16.1)
Tertiary or above	54	(11.0)	21	(4.8)	28	(6.4)	103	(7.5)
**Religion**								
Christianity	99	(21.8)	233	(48.8)	460	(92.0)	790	(55.4)
Islam	5	(1.1)	174	(36.8)	30	(6.0)	209	(14.7)
Traditional	322	(71.1)	26	(5.5)	4	(0.8)	352	(24.7)
Other religions	27	(6.0)	42	(8.9)	6	(1.2)	75	(5.2)
**National health insurance**								
No	49	(9.9)	131	(26.2)	224	(44.8)	404	(27.0)
Yes	448	(90.1)	369	(73.8)	276	(55.2)	1093	(73.0)
**Wealth index**								
Poorest	206	(45.8)	60	(12.8)	12	(2.6)	278	(20.2)
Poorer	94	(20.9)	150	(32.0)	30	(6.5)	274	(19.9)
Poor	50	(11.1)	127	(27.1)	99	(21.6)	276	(20.0)
Less poor	39	(8.7)	80	(17.0)	156	(34.0)	275	(20.0)
Least poor	61	(13.5)	52	(11.1)	167	(35.3)	275	(19.9)
**Preferred pregnancy timing**								
When pregnancy occurred	328	(66.0)	331	(66.2)	216	(43.2)	875	(58.5)
Later date	147	(29.6)	127	(25.4)	207	(41.4)	481	(32.1)
Not at all	22	(4.4)	42	(8.4)	77	(15.4)	141	(9.4)
**ANC attendance**								
≥ 4 visits	457	(92.0)	417	(83.4)	415	(83.0)	1289	(86.1)
≤ 3 visits	40	(8.0)	83	(16.6)	95	(17.0)	208	(13.9)

Notes: JHS: junior high school; JSS: junior secondary school; SHS: senior high school; SSS: senior secondary school.

In addition, 73% of women possessed health insurance, and 61% were married. Almost 75% of women were supervised by skilled attendants during birth, and 86% had attended ANC at least four times.

### Factors associated with A.N.C attendance across study sites

Table 2 shows the results of regression analysis for ANC attendance across all three sites. Cohabiting women and unmarried women (i.e. single, divorced, or widowed) were less likely to have attended ANC at least four times relative to married women (adjusted odds ratios [AOR]: 0.57; 95% CI: 0.34–0.97 and AOR: 0.39; 95% CI: 0.22–0.69, respectively). Women whose partners were educated to middle, junior secondary (JSS), or junior high school (JHS) levels were more likely to have attended ANC at least four times relative to those whose partners were uneducated (AOR: 1.64; 95% CI: 1.02–2.64). Women with national health insurance were more likely to have attended ANC at least four times (AOR: 1.67; 95% CI: 1.14–2.38) relative to those without insurance.

**Table 2.** Regression analysis results for ANC attendance across all study sites.

Characteristics	OR	(95% CI)	AOR	(95% CI)
**Geographical location**				
Navrongo	**2.34**	(1.57–3.49)***	1.91	(0.81–4.47)
Kintampo	1.03	(0.74–1.43)	1.38	(0.82–2.33)
Dodowa	**1**		**1**	
**Age group**				
15–24	**1**		**1**	
25–34	1.24	(0.90–1.72)	0.98	(0.66–1.47)
35–49	1.18	(0.77–1.80)	1.21	(0.71–2.08)
**Marital status**				
Married	**1**		**1**	
Cohabiting	**0.48**	(0.35–0.67)***	**0.57**	(0.34–0.97)*
Divorced/widowed/never married	**0.39**	(0.26–0.58)***	**0.39**	(0.22–0.69)***
**Education**				
None	**1**		**1**	
Primary	1.05	(0.74–1.50)	0.82	(0.52–1.27)
Middle/JSS/JHS	**1.76**	(1.21–2.57)**	1.26	(0.77–2.05)
Secondary and above	**4.43**	(1.76–11.15)**	1.92	(0.68–5.42)
**Partner’s education**				
None	**1**		**1**	
Primary	1.20	(0.76–1.90)	1.22	(0.72–1.08)
Middle/JSS/JHS	**1.59**	(1.08–2.32)**	**1.64**	(1.02–2.64)*
Secondary/SSS/SHS	**1.89**	(1.15–3.11)**	1.29	(0.70–2.38)
Tertiary or above	**5.44**	(1.94–15.22)**	1.76	(0.57–5.43)
**Religion**				
Traditional	**1**		**1**	
Christianity	**0.66**	(0.45–0.98)*	1.16	(0.54–2.52)
Islam	**0.57**	(0.34–0.93)*	0.72	(0.31–1.70)
Other religions	0.54	(0.27–1.08)	0.71	(0.29–1.70)
**National health insurance**				
No	**1**		**1**	
Yes	**2.10**	(1.55–2.85)***	**1.64**	(1.14–2.38)**
**Wealth index**				
Poorest	**1**		**1**	
Poorer	0.80	(0.50–1.29)	0.98	(0.56–1.72)
Poor	**0.57**	(0.36–0.90)*	0.89	(0.50–1.60)
Less poor	0.93	(0.57–1.51)	1.44	(0.74–2.79)
Least poor	**1.80**	(1.02–3.17)*	2.00	(0.94–4.26)
**Preferred pregnancy timing**				
When pregnancy occurred	**2.30**	(1.46–3.63)***	1.73	(0.99–3.03)
Later date	1.24	(0.78–1.98)	1.23	(0.71–2.12)
Not at all	**1**		**1**	

Notes: Bold values are significant (*p < 0.05; **p < 0.01; ***p < 0.001).

AOR: adjusted odds ratio; CI: confidence interval; JHS: junior high school; JSS: junior secondary school; OR: odds ratio; SHS: senior high school; SSS: senior secondary school.

### Factors associated with A.N.C attendance at each study site

Table 3 presents the results of the regression analysis for ANC attendance according to selected characteristics, in the Navrongo area. The results revealed that single, divorced, and widowed women were less likely to have attended ANC at least four times relative to married women (AOR: 0.29; 95% CI: 0.09–0.95).

**Table 3.** Regression analysis results for ANC attendance in Navrongo.

Characteristics	OR	(95% CI)	AOR	(95% CI)
**Age group**				
15–24	**1**		**1**	
25–34	2.02	(0.97–4.20)	2.38	(0.92–5.82)
35–49	1.22	(0.51–2.90)	1.50	(0.51–4.35)
**Marital status**				
Married	**1**		1	
Cohabiting	0.53	(0.06–4.46)	0.20	(0.01–2.94)
Divorced/widowed/never married	**0.31**	(0.13–0.77)*	**0.29**	(0.09–0.95)*
**Education**				
None	**1**			
Primary	1.00	(0.47–2.17)	0.88	(0.37–2.13)
Middle/JSS/JHS	1.62	(0.68–3.82)	1.73	(0.57–5.24)
Secondary and above	2.71	(0.60–12.28)	2.25	(0.34–15.0)
**Partner’s education**				
None	**1**		**1**	
Primary	1.98	(0.76–5.11)	1.80	(0.64–5.01)
Middle/JSS/JHS	2.23	(0.86–5.75)	2.46	(0.83–7.26)
Secondary/SSS/SHS	1.96	(0.71–5.43)	1.29	(0.35–4.72)
Tertiary or above	3.59	(0.81–15.9)	2.08	(0.35–12.5)
**Religion**				
Traditional	**1**		**1**	
Christianity	1.86	(0.70–4.94)	1.42	(0.30–6.73)
Other religions	0.57	(0.18–1.76)	0.52	(0.15–1.85)
**National health insurance**				
No	**1**		**1**	
Yes	1.70	(0.68–4.28)	1.21	(0.41–3.58)
**Wealth index**				
Poorest	**1**		**1**	
Poorer	0.57	(0.26–1.27)	0.47	(0.20–1.11)
Poor	0.52	(0.20–1.33)	0.41	(0.13–1.32)
Less poor	3.20	(0.41–24.86)	1.93	(0.81–20.6)
Least poor	2.48	(0.56–11.12)	0.90	(0.11–7.71)
**Preferred pregnancy timing**				
Not at all	**1**		**1**	
When pregnancy occurred	1.33	(0.29–6.03)	0.52	(0.05–5.54)
Later date	0.88	(0.19–4.14)	0.56	(0.05–6.03)

Notes: Bold values are significant (*p < 0.05; **p < 0.01; ***p < 0.001).

AOR: adjusted odds ratio; CI: confidence interval; JHS: junior high school; JSS: junior secondary school; OR: odds ratio; SHS: senior high school; SSS: senior secondary school.

Table 4 shows the results of the regression analysis for ANC attendance in the Kintampo area. Single, divorced, and widowed women were less likely to have attended ANC at least four times relative to married women (AOR: 0.37; 95% CI: 0.16–0.87), and women with insurance were more likely to have attended ANC at least four times relative to those without insurance (AOR: 2.03; 95% CI: 1.08–3.81). Women whose partners were educated to middle, junior secondary (JSS), or junior high school (JHS) levels were more likely to have attended ANC at least four times relative to those whose partners were uneducated (AOR: 2.66; 95% CI: 1.11–6.33). Relative to the poorest participants, those in the poorer (AOR: 2.69; 95% CI: 1.14–6.36) and least poor (AOR: 6.48; 95% CI: 1.26–33.5) groups were more likely to have attended ANC at least four times.

**Table 4.** Regression analysis results for ANC attendance in Kintampo.

Characteristics	OR	(95% CI)	AOR	(95% CI)
**Age group**				
15–24	**1**		**1**	
25–34	1.09	(0.63–1.88)	1.11	(0.51–2.38)
35–49	0.91	(0.48–1.71)	1.42	(0.53–3.75)
**Marital status**				
Married	**1**		**1**	
Cohabiting	1.25	(0.62–2.53)	1.05	(0.41–2.68)
Divorced/widowed/never married	**0.46**	(0.26–0.80)**	**0.37**	**(0.16**–0.87)*
**Education**				
None	**1**		**1**	
Primary	1.01	(0.55–1.84)	0.59	(0.25–1.40)
Middle/JSS/JHS	1.46	(0.81–2.60)	0.87	(0.34–2.21)
**Partner’s education**				
None	**1**			
Primary	1.97	(0.65–5.95)	2.56	(0.75–8.75)
Middle/JSS/JHS	**1.85**	**(1.01**–3.39)*	**2.66**	(1.11–6.33)*
Secondary/SSS/SHS	**3.00**	**(1.21**–7.45)**	2.48	(0.80–7.71)
Tertiary or above	5.63	(0.73–43.3)	3.30	(0.36–30.7)
**Religion**				
Traditional	**1**		**1**	
Christianity	1.80	(0.67–4.81)	2.25	(0.70–7.20)
Islam	1.44	(0.53–3.89)	1.92	(0.57–6.40)
Other religions	1.28	(0.39–4.21)	1.90	(0.47–7.75)
**National health insurance**				
No	**1**		**1**	
Yes	**2.15**	**(1.32**–3.52)**	**2.03**	**(1.08**–3.81)*
**Wealth index**				
Poorest	**1**		**1**	
Poorer	**2.25**	(1.11–4.55)**	**2.69**	**(1.14**–6.36)*
Poor	1.94	(0.95–3.95)	2.15	(0.92–5.07)
Less poor	**3.38**	(1.39–8.20)**	2.39	(0.82–6.96)
Least poor	**10.71**	**(2.35**–48.86)**	**6.48**	**(1.26**–33.5)*
**Preferred pregnancy timing**				
When pregnancy occurred	1.99	(0.91–4.32)	2.55	(0.91–7.12)
Later date	1.10	(0.48–2.52)	1.56	(0.53–4.57)
Not at all	**1**		**1**	

Notes: Bold values are significant (*p < 0.05; **p < 0.01; ***p < 0.001).

AOR: adjusted odds ratio; CI: confidence interval; JHS: junior high school; JSS: junior secondary school; OR: odds ratio; SHS: senior high school; SSS: senior secondary school.

Table 5 shows the results of the regression analysis for ANC attendance in the Dodowa area. Women who had wanted to become pregnant when their pregnancies occurred were more likely to have attended ANC at least four times relative to those who had not wanted to become pregnant at all (AOR: 2.62; 95% CI: 1.15–5.98).

**Table 5.** Regression analysis results for ANC attendance in Dodowa.

Characteristics	OR	(95% CI)	AOR	(95% CI)
**Age group**				
15–24	**1**		**1**	
25–34	1.15	(0.69–1.91)	0.69	(0.36–1.32)
35–49	1.79	(0.81–3.97)	1.18	(0.46–3.04)
**Marital status**				
Married	**1**		**1**	
Cohabiting	**0.34**	**(0.17**–0.67)**	0.46	(0.19–1.11)
Divorced/widowed/never married	0.42	(0.18–1.02)	0.54	(0.17–1.72)
**Education**				
None	**1**		**1**	
Primary	0.88	(0.50–1.56)	0.84	(0.42–1.68)
Middle/JSS/JHS	**2.03**	**(1.10**–3.75)*	1.63	(0.78–3.41)
Secondary and above	3.25	(0.95–11.07)	1.48	(0.35–6.24)
**Partner’s education**				
None	**1**		**1**	
Primary	0.66	(0.32–1.38)	0.57	(0.24–1.35)
Middle/JSS/JHS	1.50	(0.77–2.91)	0.74	(0.33–1.68)
Secondary/SSS/SHS	1.39	(0.62–3.11)	0.63	(0.23–1.73)
Tertiary or above	7.15	(0.91–56.2)	1.22	(0.13–11.6)
**Religion**				
Traditional	**1**		**1**	
Christianity	1.63	(0.17–15.9)	2.67	(0.78–9.19)
Other religions	1.67	(0.07–37.7)	0.52	(0.15–1.85)
**National health insurance**				
No	**1**		**1**	
Yes	1.57	(0.98–2.51)	1.52	(0.87–2.65)
**Wealth index**				
Poorest	**1**		**1**	
Poorer	0.66	(0.12–3.74)	0.87	(0.12–5.64)
Poor	0.53	(0.11–2.59)	0.76	(0.14–4.04)
Less poor	0.91	(0.19–4.40)	1.12	(0.21–5.99)
Least poor	1.71	(0.34–8.44)	1.78	(0.32–10.01)
**Preferred pregnancy timing**				
When pregnancy occurred	**2.44**	**(1.24**–4.80)**	**2.62**	(1.15–5.98)*
Later date	1.16	(0.62–2.17)	1.26	(0.62–2.54)
Not at all	**1**		**1**	

Notes: Bold values are significant (*p < 0.05; **p < 0.01; ***p < 0.001).

AOR: adjusted odds ratio; CI: confidence interval; JHS: junior high school; JSS: junior secondary school; OR: odds ratio; SHS: senior high school; SSS: senior secondary school.

In Table 6, we compared the proportions of participants who had attended ANC at least four times between those with and without health insurance according to age, educational level, partner’s educational level, and wealth. Among the youngest (*p* = 0.02), least educated (*p* < 0.01), and poorest (*p* = 0.01) women and women whose partners were uneducated (*p* < 0.01), those with health insurance were more likely to have attended ANC at least four times relative to those without health insurance.

**Table 6.** ANC attendance according to health insurance status in women of low socioeconomic status.

	**Overall**		**With insurance**		**Without insurance**		
	n	%	n	%	n	%	*p*-value^†^
**Age group**							
15–24	427	(84.4)	329	(86.6)	98	(77.8)	0.020
**Marital status**							
Cohabiting	321	(81.3)	183	(83.9)	138	(78.0)	0.130
Divorced/widowed/never married	149	(77.6)	118	(80.3)	31	(68.9)	0.110
**Education**							
None	483	(82.7)	325	(86.9)	158	(75.2)	< 0.010
**Partner’s education**							
None	355	(82.0)	259	(87.2)	96	(70.6)	< 0.010
**Wealth index**							
Poorest	242	(87.1)	194	(89.8)	48	(77.4)	0.010

^†^ *p*-value for chi-square test.

## Discussion

The results revealed that 86% of participants had attended ANC at least four times. The determinants of attending ANC at least four times across all three study sites included partner’s educational level, marital status, and possession of health insurance. In addition, among women with low socioeconomic status (i.e. the youngest, least educated, and poorest women and women whose partners were uneducated), those with health insurance were more likely to have attended ANC at least four times relative to those without health insurance. At a site-specific level, the following factors were identified as determinants of attending ANC at least four times: marital status in Navrongo; marital status, possession of national health insurance, partners’ educational level, and wealth in Kintampo; and preferred pregnancy timing in Dodowa.

Having a partner with a high educational level was positively associated with ANC attendance, particularly in Kintampo. In some parts of Ghana, men, as the heads of their families, hold the most important roles and responsibilities, and have unlimited decision-making power [13,14]. These men make important decisions, including those concerning whether their wives seek skilled care [12–16]. Therefore, men’s education could play a critical role in improving maternal health in Ghana. The findings corroborate the results of a study conducted in Ethiopia, in which women with partners who had completed middle or higher education were more likely to use maternal health services relative to those whose partners had completed only primary school or received no education [34]. Other studies conducted in developing countries, such as Sudan and Ghana, have also shown that low ANC attendance was associated with low educational levels of spouses [9,35,36].

Women who were divorced, widowed, or never married were less likely to have attended ANC at least four times relative to those who were married, particularly in Navrongo and Kintampo. In Ghanaian society, unmarried women are expected to remain chaste until marriage [37]; therefore, those who are pregnant could be more likely to avoid ANC services for fear of public ridicule. In addition, women who do not have partners could experience financial difficulty that might prevent them from attending ANC regularly. Moreover, the results showed that the poorest women experienced challenges in accessing ANC, particularly in Kintampo.

In Dodowa, women who had wanted to become pregnant were more likely to have attended ANC at least four times relative to those who did not want to become pregnant at all. This suggests that women with unwanted pregnancies could have been unwilling to seek or attend ANC. Although Dodowa is relatively close to the capital of Accra [38], some communities in the area are located far from health facilities. For instance, Dodowa has only 20 CHPS compounds, while the Navrongo area covers 36 [38]. Therefore, in the Dodowa area, women who lived long distances from a health facility but experienced unwanted pregnancies could have been reluctant to walk to health facilities for ANC services. In addition, absence of a pregnancy ‘mindset,’ which is common in unexpected or unwanted pregnancy, could have exerted a negative influence on women’s use of ANC services [7,39].

Women with health insurance were more likely to have attended ANC at least four times relative to their uninsured counterparts across Ghana. In general, women with low socioeconomic status commonly show poor care-seeking behavior and health outcomes [40,41]. However, we found that they were more likely to have attended ANC at least four times if they had enrolled in the health insurance scheme. Consistent with those of earlier studies [4,36,42–49], the findings of the current study demonstrated the importance of health insurance in seeking and receiving healthcare. Implementation of the Ghanaian government policy for free medical care for pregnant women under the NHIS could have been a factor in increasing ANC attendance.

However, the NHIS registration system is associated with some challenges. The ANC services are free only in public health facilities. In addition, when unregistered women visit the health facilities to seek maternal health services, they initially receive care but are required to register to avoid payment for subsequent services [50]. However, the insurance registration system is associated with long queues, making it difficult for clients to register on time [50–52]. Moreover, client registration was previously decentralized and performed at a community level, but it is now limited to district capitals because of the biometric system used; therefore, many people, including pregnant women, are required to travel long distances to district capitals to register [53].

## Study limitations

This study was subject to several limitations. First, recall bias could have limited the validity of the data, because some participants could have forgotten about past events involving ANC services. To minimize recall bias, we crosschecked self-reported information against the Maternal Health Records book, in which ANC attendance was recorded by health workers. In addition, differences in recruitment methods and training for fieldworkers and supervisors between the three different HDSS sites could have influenced interview standardization. However, study procedures and training protocols were designed to ensure that standards were met with respect to recruiting and training fieldworkers and supervisors at the three sites. Furthermore, reasons for the variations in insurance status among the three sites need to be examined.

## Conclusions

Ghanaian women who were economically disadvantaged were less likely to have attended ANC at least four times during pregnancy if they were not enrolled in the NHIS. This scheme should include all women with low socioeconomic status, to improve access to ANC in predominantly rural communities. This could be achieved by enrolling pregnant women and nursing mothers in the insurance scheme at a health-facility level. The National Insurance Authority could introduce a program to ensure that pregnant women are provided with the opportunity to register in time to receive maternal health services. Health professionals should educate pregnant women of low socioeconomic status about the benefits of the insurance scheme and maternal health services. In addition, the GHS should continue to improve their services to gain communities’ trust with respect to the health system. Finally, communities should be encouraged to establish support systems to assist socioeconomically vulnerable women in accessing maternal health services.

## Paper context

Ghana has introduced free maternal health service under the National Health Insurance Scheme in 2004. It contributed to improve access to antenatal care (ANC), but low socioeconomic women had limited access to the scheme and were less likely to achieve at least four ANC visits. To increase coverage, insurance registration system should be improved and thereby improve their ANC visits.
